# Optimal sandwich panel's core design for an enhanced impact resistance

**DOI:** 10.1016/j.heliyon.2024.e41211

**Published:** 2024-12-18

**Authors:** Assil Charkaoui, Noha M. Hassan, Zied Bahroun

**Affiliations:** a Materials Science and Engineering Program, College of Arts and Sciences, American, University of Sharjah, P.O. Box 26666, Sharjah, United Arab Emirates; b Department of Industrial Engineering, College of Engineering, American University of Sharjah, P.O. Box 26666, Sharjah, United Arab Emirates

**Keywords:** Sandwich panels, Energy absorption, Additive manufacturing, Impact resistance, Core geometry, Optimization

## Abstract

Despite the extensive literature revealing various core structures that can enhance the impact resistance of composite panels, a comparative study illustrating the difference in performance of the various cores under same loading conditions is missing. The aim of this study is to determine the optimal core structure and design in terms of energy absorption under low-velocity impact using both numerical simulations and experimental testing for validation. Response surface analysis was used to design the experiments and analyse the panel's behaviour. A total of 160 numerical simulations were conducted by varying the core shape, density, number of layers and panel's thickness. Drop tower tests were performed to experimentally validate the results. Additive manufacturing was used to 3D print the tested structures which simplified the manufacturing process. Results provided insights on time to perforation, stiffness of the panels showcasing an inverse relationship between recoverable strain energy and equivalent plastic strain. The number of layers within the panels were identified as a pivotal factor in the energy distribution and tendency for localized plastic deformation to occur. Both numerical and experimental results revealed the superior energy absorption capabilities of the X-frame core shaped structure. Regression models developed shed light on the relationships between core height, volume fraction, number of layers, and core topology revealing the extent of damage. Multi-objective optimization was used to yield optimal configuration for sandwich panels with highest impact resistance.

## 1 Introduction

Sandwich panels, known for their lightweight versatility, are gaining attention in various industries. These panels offer an impressive combination of strength and low weight, making them suitable for diverse applications, including impact mitigation, indentation resistance, and heavy load support [1,2]. Therefore, the automotive and aerospace sectors are showing increased interest in sandwich structures due to their lightweight nature and excellent impact resistance. In recent years, significant research has focused on sandwich panels with thin outer layers and high-porosity cellular cores, highlighting their outstanding strength-to-weight ratio and resilience to impacts, especially in out-of-plane scenarios [3]. Typically, sandwich structures consist of two thin outer layers made from materials like fiberglass [4], carbon fibre composites [5], or aluminium [6], enveloping a core material such as foam, honeycomb, or lattice truss [7]. This unique blend of rigid outer layers and flexible core materials enhances specific bending strength and stiffness, making sandwich structures a subject of intensive investigation. The outer layers play a crucial role in supporting in-plane and bending loads, while the core excels in resisting shear forces and lateral compression [8]. Additionally, the core contributes to enhancing bending stiffness and improving the energy absorption capabilities of composite structures [9]. The heightened interest in sandwich structures has prompted extensive research to enhance their mechanical properties. The performance of sandwich panels largely depends on the characteristics of their core and outer layers. Numerous studies have explored the influence of outer layer properties on sandwich panels [[10], [11], [12]]. In different efforts to improve sandwich panel performance, conventional approaches involve adjusting size parameters or core materials [13,14]. Additionally, modifying or reshaping the core's geometry offers even greater flexibility. Various types of sandwich cores have been developed, including the conventional hexagonal honeycomb core (HHC), auxetic honeycomb core [15], foam core [8], corrugated core [16], among others.

Sharei et al. [17] examined the low-velocity impact response of foam core sandwich panels reinforced with carbon, aramid, and carbon-aramid hybrid short fibres, showing that fibre reinforcement significantly improves mechanical properties by 18–30 % compared to non-reinforced foam. Numerical simulations using ABAQUS closely matched experimental results with a 9.1 % average difference, demonstrating the effectiveness of fibre reinforcement. The use of 3D printing has been dominant in the field of cellular material and various studies have been done to investigate the effect of varying the cell shapes. In a study by Montazeri et al. [18], investigates six 3D-printed honeycomb structures with varying Poisson's ratios using three-point bending tests and finite element analysis, revealing that hybrid hexagonal and re-entrant unit honeycombs significantly outperform conventional designs in load-carrying capacity and energy absorption. The hybrid geometry honeycombs exhibit superior performance, absorbing up to 475.1 % more energy, making them promising for automotive, protective, and construction applications.

In addition to the exploration of geometrical effects, research has also focused on the manufacturing processes of composite structures, such as in vacuum infusion moulding. In a study by Shevtsov et al. [19], vacuum infusion moulding was employed to manufacture thin-walled polymer-composite structures, examining various process control modes. The study highlighted how factors such as resin propagation, fiber volume fraction, and process conditions significantly influence the mechanical properties of composite materials. The use of optimized vacuum infusion moulding techniques allows for better fiber distribution, which improves structural integrity and mechanical performance. This aligns with the present study's focus on optimizing the topological design of sandwich structures to enhance their energy absorption and load-bearing capabilities.

Taghizadeh et al. [20] studied how corrugated geometry affects the energy absorption of composite sandwich panels. They found that the core geometry has a significant effect on the mechanical properties and failure modes in which corrugated cores showed better energy absorption. Moreover, the corrugation angles influenced core crushing resistance and panel deflection. Ha et al. [21] presented a novel woodpecker-inspired sandwich panel, showing superior energy absorption capacity compared to the standard honeycomb pane. The bio-inspired panel achieved 125 % higher specific energy absorption and a 63.7 % improvement with equivalent core volume. Haq et al. [22] improved blast mitigation in sandwich panels by optimizing energy absorption using Finite Element Method simulations. They introduced an efficient configuration by combining AuxHex and Star-Reentrant honeycomb core shapes, which outperformed traditional hexagonal cores with 28 % and 19.2 % higher impact energy absorption, respectively. Sun et al. [23] used finite element analysis to study sandwich panels with hierarchical honeycomb cores under impulse loading. They examined how different structural parameters, including cell wall thickness and core height affected blast response. The regular honeycomb core had lower deflection for low-impulse loading. Thinner cell-wall thickness enhanced resistance at low impulses, but the trend reversed at high impulses. The core height of 30 mm had the lowest specific energy absorption but the highest blast resistance. Sadiq et al. [24] investigated sandwich panels with honeycomb cores. They used finite element modelling and experiments to study transient responses and examined core height, cell size, and cell wall thickness. Increasing core height from 5 to 20 mm reduced the maximum transient response by about 82.78 %. Although the core height had the most significant impact on the transient response, varying the cell wall thickness also increased the maximum transient response. Cell wall thickness had a smaller effect in comparison. The investigation varying the cellular core within sandwich panels and assessing them using numerical tools has been also studied by Sefidi et al. [25] by fabricating expanded metal mesh cores from ASTM A-611 high-strength steel and integrating them into sandwich panels with steel face sheets. The study used numerical simulations and analytical models to evaluate the mechanical properties of the sandwich beams under quasi-static three-point bending, focusing on energy absorption and the formation of plastic hinges at cell nodes. The results showed that the failure modes were influenced by core geometry, with face yielding dominating in smaller core sizes and indentation core failure observed in larger core sizes. Variations in core strand thickness and the ratio of core dimensions significantly affected the sandwich panel's compressive and shear strengths. Adding on, Taghipoor et al. [26] has experimentally investigated the impact of hole size on the crushing force of square thin-wall tubes under high strain axial impact, showing that while increasing tube length does not significantly affect the maximum force, the presence of holes slightly reduces the average and maximum forces and decreases collapse force efficiency (CFE) by up to 46 %, with larger hole diameters further reducing the maximum force. This response can be similar to that of cellular cores within sandwich panels.

Charkaoui et al. [27] provides an extensive review of the literature illustrating how core design, height, cell type, layering, and grading significantly affects structural performance and multifunctionality in different applications. The existing literature reveals a notable gap in research pertaining to the concurrent examination of different core structures under same loading condition to comparatively assess their performance. In addition, considering multiple geometrical factors at the same time to determine the interrelation between those factors on the overall performance of the composite panel. To address this, the present study conducted a comprehensive numerical investigation on sandwich panels featuring distinct core geometries, including x-frame, I-frame, rhombus, and H-frame configurations.

The selection of the X-frame, I-frame, rhombus, and H-frame core geometries for this study was based on a combination of practical and functional considerations. First, these geometries were chosen for their ease of design and manufacturability using 3D printing technology, specifically fused deposition modeling (FDM). This was essential for maintaining consistency in the fabrication process, especially when varying critical parameters such as core height, volume fraction, and the number of layers. Furthermore, each of the selected geometries offers distinct structural advantages. The X-frame core excels in energy absorption due to its ability to effectively distribute loads and resist buckling, making it ideal for high-energy impacts. The I-frame provides high bending stiffness, particularly in directions parallel to its central axis, enhancing its ability to resist out-of-plane bending loads. The rhombus geometry offers a balance between stiffness and flexibility, making it well-suited for moderate energy absorption while maintaining structural integrity. Lastly, the H-frame core, while more traditional, serves as a simple structure that provides basic load-bearing capacity and offers a useful benchmark for comparison with the more complex geometries.

Additionally, these core geometries have been studied extensively in previous research, particularly in the context of energy absorption and impact resistance. Their inclusion in this study not only provides a point of comparison with other established designs, such as honeycomb or auxetic cores, but also allows for benchmarking and validation against prior findings.

Various cellular core volume fraction (ranging from 10 % to 20 %), height (10 mm, 20 mm, 30 mm), and number of layers (1, 2, 3) was considered. The study employed Design of Experiments (DOE) techniques to systematically examine these factors and their interaction. Numerical experiments were then conducted to study the panel's behaviour under low-velocity impact. Results were then validated experimentally using a drop tower test.

## 2 Materials and methods

### 2.1 Numerical methodology

**Fig. 1:**
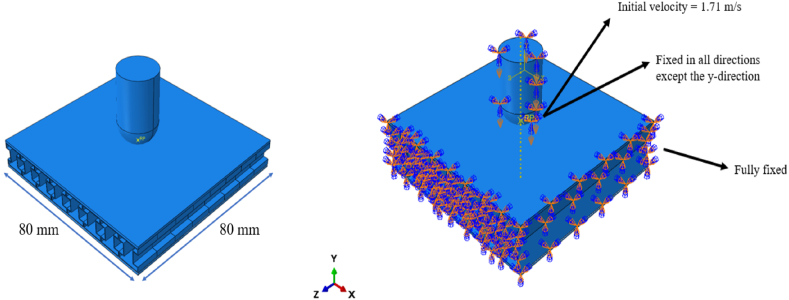
(a) Sandwich panel with impactor; (b) Load and boundary conditions of structure.

The low-velocity impact analysis was modelled using Abaqus/Explicit software to simulate the low velocity penetration of a sandwich panel accounting for high non-linear deformations and strain rate. An FE model consisting of the sandwich panel, impactor and target fixture was modelled, as shown in Fig. 1a, in accordance with the experimental setup of the drop tower impact test. The simulation parameters used in Abaqus for the low-velocity impact tests were designed in accordance with industry standards for impact resistance testing, specifically following the guidelines of ASTM D1596. This standard provides the recommended procedures for assessing the impact behavior of materials under low-velocity conditions, ensuring that the testing methodology is both consistent and reliable.

In accordance with ASTM D1596, the impact velocity, boundary conditions, and material properties were carefully selected to replicate real-world impact scenarios accurately. For example, the drop height and impactor mass were calculated based on the standard's requirements for potential energy conversion into kinetic energy. Additionally, the fixture setup for the sandwich panels in the simulation mirrored the constraints outlined in the ASTM standard to ensure realistic force and deformation responses during the impact event. By adhering to ASTM D1596, the study ensures that the simulation parameters reflect industry-recognized impact resistance testing practices, making the results both valid and comparable to experimental tests used in industry.

The face sheets and core of sandwich panels as well as the impactor were meshed using the 8-node solid elements with reduced integration (C3D8R) to account for out-of-plane mechanical behaviour.

**Fig. 2:**
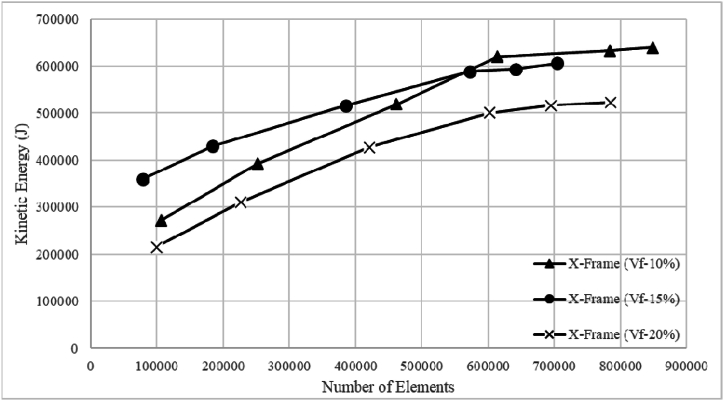
Mesh sensitivity analysis for varying topological features.

A mesh sensitivity analysis was conducted to refine the mesh ensuring accurate results and reasonable computational time as shown in Fig. 2. Since the topological features have direct influence on the volume fraction, the mesh analysis was conducted on varying the volume fraction only and the mesh size was kept constant for all the simulations. The convergence of kinetic energy results with a mesh comprising 613,512 elements, demonstrating a marginal 2 % difference compared to a denser mesh of 784,132 elements. This minor discrepancy suggests that increasing the number of elements beyond 613,512 does not significantly enhance accuracy, thereby establishing 613,512 elements as the threshold for result convergence. To achieve this element count, a mesh size of 0.12 mm is used for the simulations.

The sandwich panel, both core and face sheets, was assumed to be made out of aluminium, and defined using the Johnson-Cook model properties given in Table 1. The impactor, on the other hand, was modelled as a rigid body. To emulate real-life impact scenarios, a reference point was established on the impactor, which served as the point of application for the impact velocity. The same material was used across all simulation runs to focus the study on the effect of the other design attributes considered.

**Table 1:** Material Properties [28].

Johnson-Cook Parameter	Aluminium (Al6061)
Density	2.7 × 10^−6^ g/mm^3^
Young's Modulus	70000 MPa
Poisson's Ratio	0.3
Yield Stress, A	324 MPa
Strain Hardening Parameter, B	114 MPa
Strain Rate Parameter, C	0.002
Thermal Softening Exponent, m	1.34
Strain Hardening Softening, n	0.42
Damage Constant, d_1_	−0.77
Damage Constant, d_2_	1.45
Damage Constant, d_3_	0.47
Damage Constant, d_4_	0
Damage Constant, d_5_	1.6

The panel possesses dimensions of 80 mm × 80 mm × h mm and incorporates a structural core with a varying cell height and thickness. These were varied to achieve different volume fractions of 10, 15, and 20 %. However, the front and back sheets have a constant thickness of 1 mm each. The methodology employed in this study is made of a parametric investigation into the impact performance of sandwich panels. Using a Design of Experiments (DOE) approach, five key variables were varied to assess their individual and combined effects on the panels’ energy absorption and stiffness. The parameters investigated include core geometry, cell orientation, core volume fraction, core height, and number of layers. A response surface design was used to explore the variations, with the simulations providing insight into how each variable contributed to the overall performance of the panels. The parametric study systematically varied the levels of each factor to ensure a complete evaluation of the design space. Through this approach, a total of 160 simulations were performed, enabling the identification of the optimal configuration that maximize energy absorption while minimizing the structure mass.

**Fig. 3:**
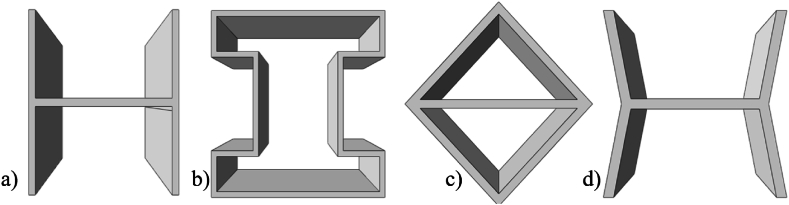
Unit cell & sandwich panel configuration of (a) I-frame; (b) rhombus; (c) X-frame; (d) H-frame.

**Fig. 4:**
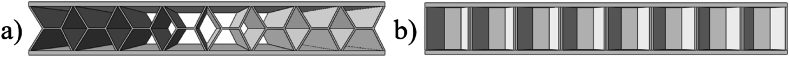
X-frame core sandwich panel with (a) transverse direction of unit cell; (b) longitudinal direction of unit cell.

**Fig. 5:**
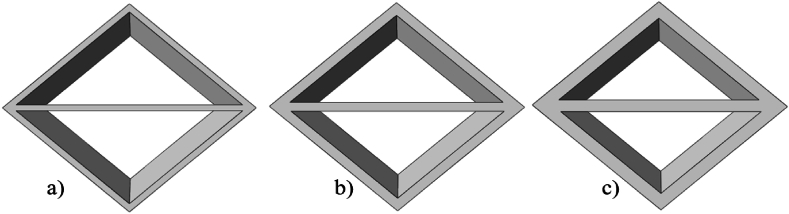
Rhombus unit cell with volume fraction of (a) 10 %; (b) 15 %; (c) 20 %.

**Fig. 6:**
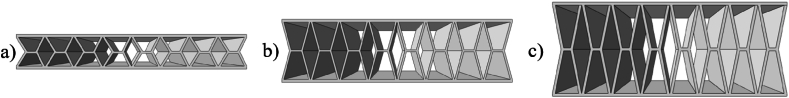
X-frame core sandwich panel with core height (a) 10 mm; (b) 20 mm; (c) 30 mm.

**Fig. 7:**
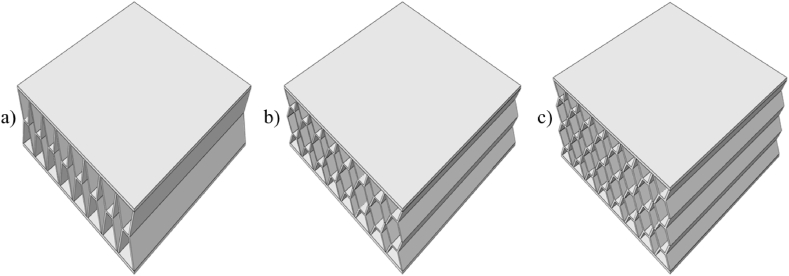
X-frame sandwich panel with (a) 1 core layer; (b) 2 core layers; (c) 3 core layers.

Table 2 gives the various levels for each of the design variables. Different unit cell structures, namely H-frame, I-frame, Rhombus, and X-frame were explored to assess their impact on panel behaviour as shown in Fig. 3. Unit cell orientation, distinguishing between transverse and longitudinal orientations was examined as portrayed in Fig. 4. The volume fraction of the core material was varied, encompassing 10 %, 15 %, and 20 % volume fractions by changing the core thickness as illustrated in Fig. 5. The relative density of the 3D-printed structures in this study is dependent on the core volume fraction. As the core volume fraction represents the percentage of solid material within the total core volume, it is directly correlated to the relative density. In this study, three different core volume fractions were considered: 10 %, 15 %, and 20 %. Therefore, these core volume fractions are representative of the relative densities of the 3D-printed structures. This variation in relative density is crucial as it impacts the mechanical properties of the sandwich panels, particularly their energy absorption capacity and stiffness, as explored in the simulations and experimental work. Unit cell height was varied with heights of 10, 20, and 30 mm as shown in Fig. 6. The number of layers in the sandwich panels considered was 1, 2, and 3 layers while keeping the core height at desired level as shown in Fig. 7.

**Table 2:** Variable of the tested sandwich panels

Factor	Levels	Values
**Geometry**	4	H-frame, I-frame, X-frame, Rhombus
**Direction**	2	Longitudinal, Transverse
**Volume Fraction**	3	10, 15, 20
**Height**	3	10, 20, 30
**Number of Layers**	3	1, 2, 3

For the purpose of comparative analysis, specific parameters in the panel configuration were held constant based on the DOE methodology employed in this study. In particular, volume fraction was systematically varied, and when set to a specific value (10 %, 15 %, or 20 %), all other parameters such as core geometry, direction, core height, and number of layers were altered while maintaining the constant volume fraction. This allowed for an isolated analysis of how changes in other variables affected panel performance under a consistent volume fraction. For example, when the volume fraction was set to 15 %, the entire set of simulations was conducted with a 15 % volume fraction, while the geometry, direction, height, and number of layers were varied. The same approach was applied for the 10 % and 20 % volume fractions. Additionally, the overall panel dimensions were kept constant across all tests to ensure uniformity and enable direct comparisons of performance based on the other changing factors. This approach allowed for a systematic investigation of the effects of each design parameter on the energy absorption and stiffness of the sandwich panels.

In terms of boundary conditions, the sandwich panel was fixed from all sides as shown in Fig. 1b. To accurately replicate the behaviour of the impactor, the impactor was fully constrained from all sides except for its vertical translational direction, which allowed for controlled movement exclusively along a specified path.

As for the loading conditions, the initial velocity of the impactor was determined based on the drop height assuming all potential energy from drop is converted to kinetic energy. By equating the KE to the PE, $\frac{1}{2}mv^{2}=mgh$, where m is the mass of the impactor, v is the velocity, g is the acceleration due to gravity, and h is the drop height, the velocity just before impact was calculated. For a consistent 15 cm drop height, the impactor's velocity just before impact was computed at 1.71 m/s. The mass of the impactor was measured to be 0.15 kg. This velocity was used as the initial velocity for the impactor positioned directly above the sandwich panel. Furthermore, the simulation was run for a total time of 0.05 s (step time) across all simulations to ensure consistency in the analysis of the impactor's penetration dynamics.

**Fig. 8:**
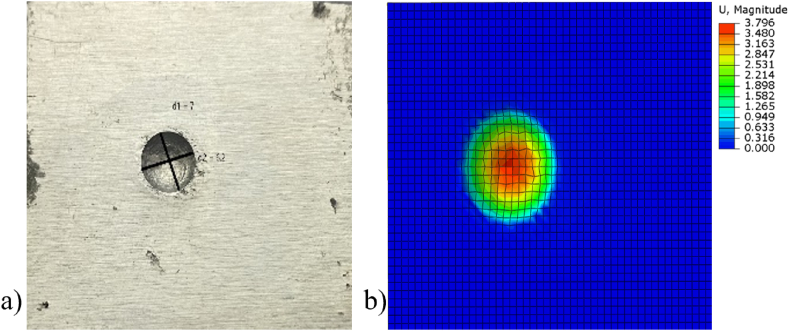
– Top view of depth of impact on an Al6061 block a) experimental b) numerical.

To validate the Abaqus simulation setup, a verification model was created and tested experimentally. This model involved subjecting an aluminium (Al6061) plate to low-velocity impact, with particular attention to the depth of the impact. This experimental approach effectively verified the accuracy of the numerical setup, confirming that it could reliably replicate the physical impact behaviour. An aluminium block with a height of 15 mm and width 43 mm was tested under low velocity impact at a speed of 2.97 m/s (corresponding to an impact height of 45 cm), both experimentally and numerically, as illustrated **in** Fig. 8.

The depth of impact for the experimental study was measured at 3.3 mm, while the numerical study recorded a depth of 3.796 mm. The depth of impact observed in the experimental verification was closely aligned with the simulation results, validating the methodology used in this study. Consequently, the validated setup was used for all the simulations, ensuring a consistent and reliable approach to modelling the low-velocity impact on different materials and configurations.

### 2.2 Experimental methodology

Before proceeding further and conducting the 160 numerical simulations, few experiments were performed numerically and compared to the experimental results. In those experiments only the core shape with varied keeping all other conditions constant. Four different core shapes were examined X-frame, H-frame, I-frame, and rhombus. For consistency, all other factors were kept constant with a volume fraction of 10 %, a core height of 10 mm, one layer, and a transverse cell direction. To simplify the fabrication process, the sandwich panels were fabricated via 3D printing using a Flashforge 3D printer, employing fused deposition modelling (FDM) technology with polylactic acid (PLA). PLA was selected due to its wide availability and ease of use, aligning with the study's emphasis on investigating topological attributes rather than the inherent material properties. The filament diameter employed was 1.75 mm.

**Fig. 9:**
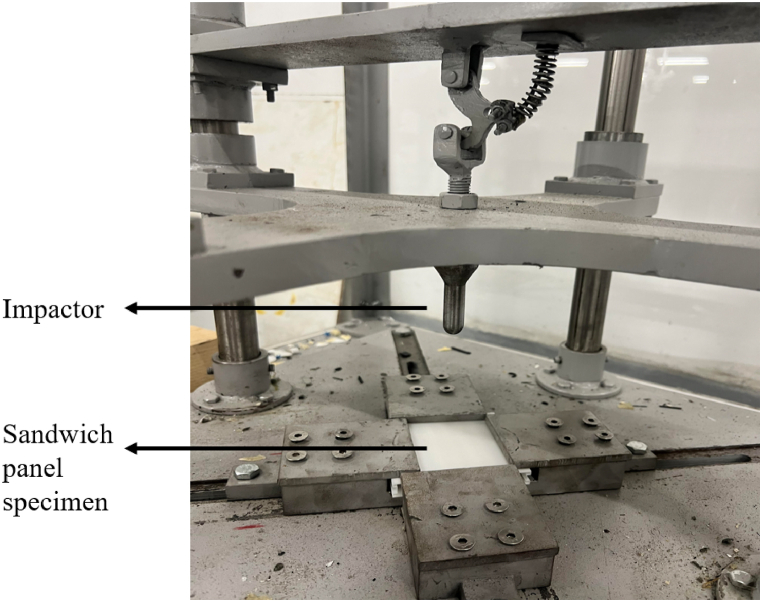
Experimental setup.

The experimental setup for conducting low-velocity impact tests on the specimens is depicted in Fig. 9 and is designed to replicate the numerical model. The impact tests were performed using a drop tower apparatus configured to follow the guidelines of ASTM Standard D1596 for impact resistance testing. In this experimental setup, a steel impactor with a sphere-shaped head and a radius of 10 mm, weighing 0.18 kg, was attached to a 15 kg guide plate to ensure a controlled, vertical free fall. The guide plate, held in place by vertical rails, was released from a height of 15 cm, resulting in an impact velocity of 1.71 m/s immediately before contact with the specimen.

The sandwich panels were securely constrained along all four edges using a rigid clamping mechanism to ensure they remained fixed during the impact. Only translational motion along the direction of the applied load was allowed, ensuring accurate force application and specimen deformation. The impactor and guide plate assembly were designed to minimize any lateral motion, ensuring that the impact force was consistently applied to the center of the panel for each test.

To ensure accurate results, each test was repeated on two identical specimens per configuration to account for any minor variances in material properties or test conditions. The damaged areas on the face sheets of the sandwich panels were measured immediately after each impact using digital callipers. The extent of damage was recorded on both the front and back face sheets to quantify the energy absorption capacity of each panel.

The damage assessment of the sandwich panels involved assuming elliptical shapes for the damage on both the front and back face sheets, characterized by major diameter (d_1_) and minor diameter (d_2_). To quantify the damage area accurately, the formula for the area of an ellipse was employed:

(1) $$Impact\;Damage\;Area=\pi \;x\;\left(\frac{d1}{2}\right)x\;\left(\frac{d2}{2}\right)$$

During the low-velocity impact tests, the specimens underwent perforation, imposing the measurement of damage areas on both the front and back face sheets.

During the experimental validation using drop tower tests, some limitations were encountered that could have affected the accuracy and consistency of the results. One notable limitation was the inherent variability in the 3D printing process used to fabricate the sandwich panels. Differences in layer adhesion, material anisotropy, and small defects that arise during 3D printing can influence the structural performance of the printed specimens, particularly under impact loads. To mitigate this, strict quality control measures were employed, including ensuring consistent printing parameters (such as extrusion temperature and layer height) and inspecting the printed specimens for defects before testing.

Another limitation was the repeatability of the drop tests. The drop tower test setup could introduce slight variations in impact angle or contact conditions, which could influence the observed damage area or energy absorption. To address this, we conducted multiple tests for each configuration (two identical samples per configuration) to ensure the results were statistically reliable. Additionally, we ensured that the impactor and sandwich panels were properly aligned and secured before each test to reduce variability in impact conditions.

**Fig. 10:**
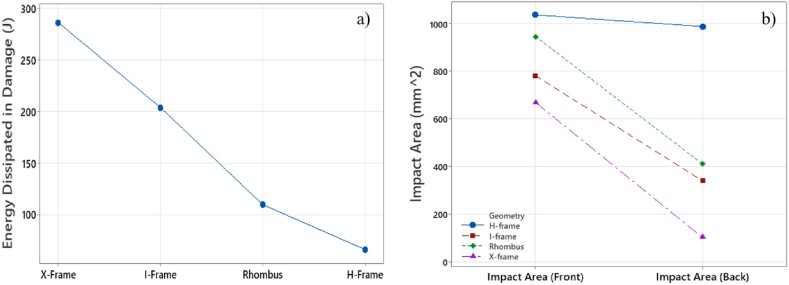
(a) Numerical assessment for energy dissipated in damage; (b) experimental assessment for damage area.

**Fig. 11:**
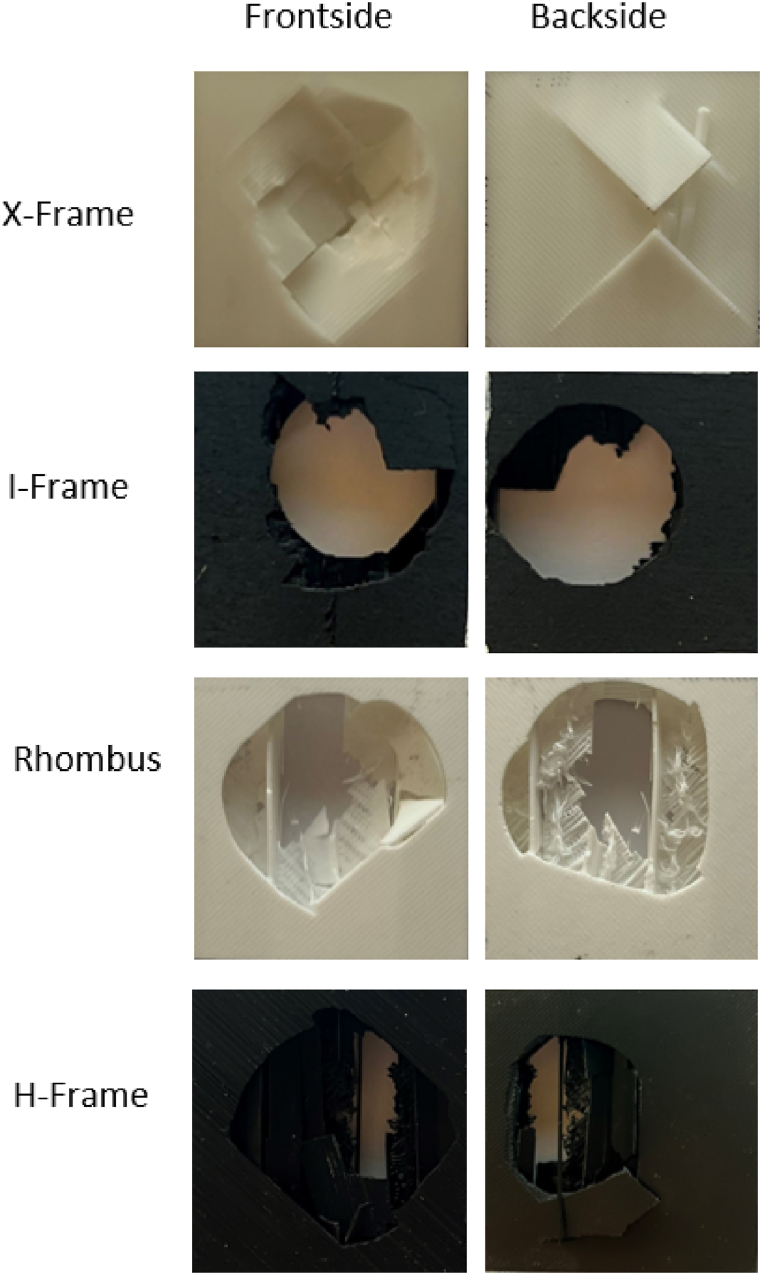
Impact damage on sandwich panels.

Fig. 10, Fig. 11 show the impact area on the front and back face sheets of sandwich panels with different core geometries. The X-frame core exhibited the smallest impact area, indicating its superior ability to absorb a higher amount of impact energy. In contrast, the H-frame core displayed the largest damage area on both the front and back face sheets, highlighting its limited energy capabilities. This is evidenced by the nearly identical damage areas on the front and back face sheets.

FromFig. 10b, the averaged values out of a total of five tested samples for each shape is shown. For the H-frame geometry a 1030 mm^2^ for the front face sheet and 982 mm^2^ for the back face sheet, where the recorded standard deviations were recorded to be ±19.2 mm^2^ and ±22.1 mm^2^ and for the front and back face sheet respectively. The second highest damage was recorded to be for the Rhombus where the average impact area was measured to be 942 mm^2^ and 408 mm^2^ for the front and back face sheet respectively with standard deviations of ±31.7 mm^2^ and ±27.1 mm^2^ for the front and back sheets respectively. As for the I-frame geometry the average impact area is measured to be 782 mm^2^ and 382 mm^2^ for the front and back face sheets with standard deviations of ±31.7 mm^2^ and ±27.1 mm^2^ for the front and back sheets respectively. Lastly, the least damage was measured for the X-frame geometry where the average impact area was measured to be 672 mm^2^ and 141 mm^2^ for the front and back face sheets with standard deviations of ±22.7 mm^2^ and ±17.7 mm^2^ for the front and back sheets, respectively.

These experimental findings align with the numerical results depicted inFig. 10a, where the X-frame core also demonstrates the highest energy dissipated in damage. This outcome indicates that the X-frame core is proficient in maximizing energy absorption. Despite the damage extending to the back face sheets, the X-frame core achieved the most efficient reduction in impact area.

Fig. 11 shows the impact damage of the sandwich panels for various core geometries, all of which maintain a constant relative density, core height, and number of layers. The low-velocity impact induces a combination of shear and out-of-plane compression forces on the sandwich panels, with slight in-plane tension forces occurring at a microstructural level. This is evident in the images, where the fibres of the PLA material appear stretched before reaching their tensile strength, leading to material fracture. Since PLA is a brittle material, there is minimal to no ductility within the core's cell walls at a microstructural level, resulting in low non-linear deformations and limiting the material's energy absorption capacity.

The face sheets of the panels initially absorb the low-velocity impact, after which the load is transferred to the unit cells, where it is distributed as flexural loads on the cells. The brittleness of the PLA results in consistent failure mechanisms across all cellular shapes. However, differences in the overall damaged area are observed as the core geometry varies. Since the constituent material, topological, and morphological features remain constant across all specimens, geometry is the only variable factor. Thus, we can conclude that the flexural modulus or yield strength of the sandwich panels is enhanced as the damaged area decreases with changes in core shape.

At the microstructural level, the tensile strength of the PLA governs the strength of the cell walls. A more consistent stress distribution can be achieved by organizing the cell walls in shorter, angular shapes supported by shorter walls, resulting in fewer fractured cells. In the X-frame, it is evident that while the face sheet is penetrated from both the top and bottom, the unit cells are not completely perforated, demonstrating better stress redistribution compared to other shapes. In contrast, the three other geometries show complete fracture in the impact area, with the impactor fully perforating the sandwich panel. The applied load from the impactor induces higher tensile forces on the unit cells, causing complete cell wall fracture.

## 3 Results & discussion

The performance of the sandwich panels under impact loads is done through a comprehensive analysis of the energy dissipation and structural integrity of the panels. The investigations were conducted using Abaqus/Explicit simulations, which provided detailed insight into the several parameters that influence the behaviour of the panels. Specifically, energy dissipated in damage and total recoverable strain energy were analysed alongside time perforation and stress distribution.

**Fig. 12:**
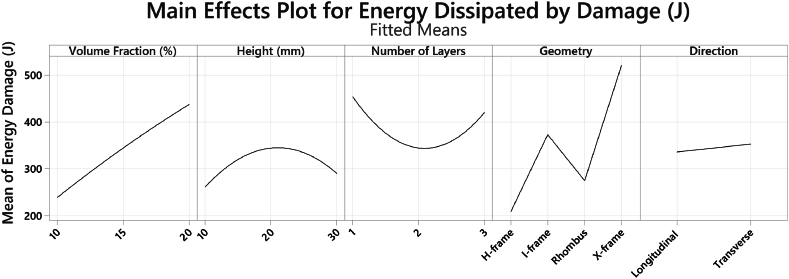
Averaged energy dissipated in damage with varying parameters.

The energy dissipated in damage was derived from “ALLDMD” values in simulations and serves as a measure for damage tolerance and impact resistance within the sandwich panels as shown in Fig. 12. Higher energy dissipated in damage indicates enhanced energy absorption capabilities. The X-frame core showed better load distribution which achieved the highest energy dissipation of 500 J. Transverse orientation of the core absorbed more energy (342 J), due to changes in impact area and inertia. Additionally, unit cells with higher volume fractions increased the energy dissipation and panel stiffness, showing a linear relationship between the volume fraction and energy damage. Increasing the height of the unit cells induced better energy absorption capabilities with optimal performance at cell height of 20 mm since it reduced the stress concentrations. However, further increasing the height of the unit cells reduced the stiffness and diminished the energy dissipation benefits. Similarly, increasing the number of core layers showed non-linear energy dissipation effects which indicates potential drawbacks. The non-linear relationship observed with increasing the number of core layers can be attributed to the complexity of stress interactions within the layered structure. As layers increase, the initial benefits of energy absorption may diminish due to stress concentrations and interference between layers, leading to less efficient energy distribution. This phenomenon is consistent with findings where increasing structural complexity in cellular materials can lead to stress concentration and reduced overall stiffness, affecting energy dissipation efficiency [29].

**Fig. 13:**
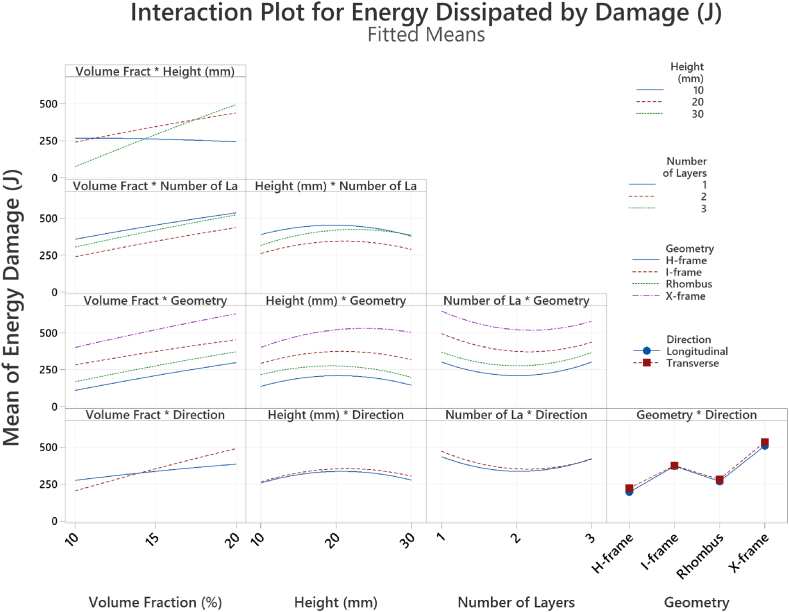
Interaction plot for energy damage.

The interaction plot in Fig. 13 shows how the levels of two factors interact and affect a response variable in the tests conducted. The energy dissipation varies with core geometry with the X-frame in the transverse direction showing superior energy absorption. Higher energy dissipation capabilities are evident for all X-frame geometries regardless of the different factors. Moreover, regardless of the volume fraction, the transverse orientation within the core exhibits higher energy dissipation as compared to the longitudinal direction. The interaction plot between the height and the volume fraction shows that the combination of taller unit cells and increased volume fraction leads to increased energy dissipation up to a certain level, after which it starts to decrease.

**Fig. 14:**
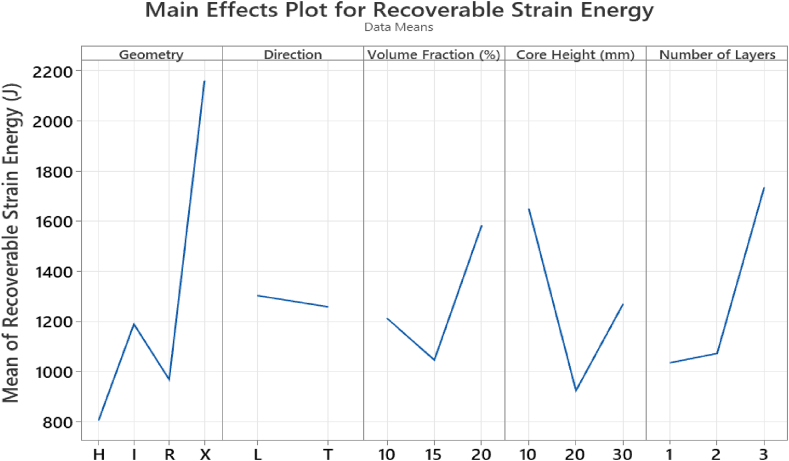
Averaged Recoverable Strain Energy with varying parameters.

“ALLSE” in Abaqus/Explicit refers to the total recoverable strain energy which indicates how much energy is stored during deformation. Fig. 14 shows that the X-frame core geometry outperforms the different geometries in recoverable strain energy (2321 J). The X-frame shows higher bending stiffness and ductility which allows it to endure higher strains before failure. While the longitudinal orientations portray higher ductility, they do not show higher energy absorption capabilities. This means that although they have good energy storing capabilities, they may not dissipate the energy in a way that minimizes the damage. Moreover, increasing the volume fraction from 10 to 20 % enhances the recoverable strain energy but can also lead to brittleness at higher volume fraction. A lower unit-cell height results in highest recoverable strain energy of 1623 J which shows that lower heights could lead to higher energy recovery. On the other hand, increasing the number of core layers increases both the recoverable strain energy and the ductility.

The variations in core volume fraction have a significant impact on both the stiffness and energy absorption properties of the sandwich panels across different geometries. Increasing the core volume fraction results in added material within the core, which enhances the overall stiffness of the panel. This is particularly evident in geometries like the X-frame and I-frame, where the increased material improves both out-of-plane stiffness and in-plane rigidity due to the geometry's ability to distribute loads more effectively. However, for simpler geometries such as the H-frame, the increase in stiffness is more limited, as the geometry does not optimize load distribution as efficiently. Furthermore, at higher volume fractions, the structure may become excessively stiff, leading to brittleness, particularly in geometries like the rhombus and H-frame, which are less capable of handling nonlinear deformations.

In terms of energy absorption, increasing the core volume fraction typically enhances the panel's ability to absorb impact energy, as the addition of material enhances the stiffness and yield stress of the sandwich panel, contributing to a bigger area under the force-displacement curve in the plateau regions. This relationship is most pronounced in geometries like the X-frame, where the increased volume fraction contributes to better load distribution and more efficient energy dissipation. The linear relationship between volume fraction and energy absorption suggests that, up to a certain point, increasing the volume fraction improves the panel's ability to handle impact loads. However, beyond a certain volume fraction, the panel can become too stiff to deform plastically, reducing its ability to absorb energy effectively. This is especially true for geometries like the H-frame and rhombus, where higher volume fractions lead to stress concentration and premature failure, diminishing the overall energy absorption efficiency.

There is often a trade-off between stiffness and energy absorption, particularly at higher volume fractions. While the X-frame continues to perform well in both stiffness and energy absorption, geometries like the I-frame and H-frame tend to suffer from reduced energy dissipation as they become more rigid. In these cases, the increased stiffness can limit the structure's ability to deform and absorb energy during impact. Therefore, for applications that prioritize energy absorption, maintaining a moderate volume fraction, such as 15 %, may provide an optimal balance between flexibility and impact resistance. On the other hand, applications requiring higher stiffness may benefit from a higher volume fraction, with the understanding that the energy absorption capacity might be compromised.

The core height of the sandwich panels plays a crucial role in determining both the load-bearing capacity and the balance between stiffness and energy absorption. As the core height increases, the load-bearing capacity generally improves due to the added distance between the outer face sheets, which enhances the panel's ability to resist bending loads and distribute forces over a larger area. This increased distance leads to improved stiffness and greater resistance to deformation, particularly under compressive and bending loads.

However, the trade-offs between stiffness and energy absorption become evident as the core height changes. While a taller core increases the stiffness of the panel, allowing it to bear higher loads, it can also lead to reduced energy absorption. This reduction occurs because the panel becomes more rigid, limiting its ability to deform plastically and dissipate energy efficiently during an impact. In contrast, shorter cores tend to enhance energy absorption due to their increased flexibility, which allows for greater plastic deformation and more efficient energy dissipation, but at the cost of lower stiffness and reduced load-bearing capacity.

An optimal core height was observed at 20 mm, where the balance between stiffness and energy absorption was most favourable. Beyond this height, the stiffness continued to increase, but the ability to absorb energy diminished due to the development of localized stress concentrations and reduced plastic deformation. It is essential to realize that the optimum core height is also dependent on the volume fraction of the core, where the stiffness of the material can vary based on the height as well as the volume fraction.

The non-linear relationship between the number of layers in the sandwich panels and their energy dissipation arises from the complex deformation mechanisms that occur as additional layers are introduced.

Initially, increasing the number of core layers enhances the panel's ability to dissipate energy. This is because additional layers provide more material to absorb impact energy and redistribute stresses, which leads to a greater resistance to deformation and improved energy absorption. In particular, the presence of multiple layers creates redundancy in the load paths, enabling the structure to manage localized failures more effectively without compromising the panel's overall performance. As a result, the energy dissipation increases with the addition of layers, up to a certain point.

However, this trend becomes non-linear as the number of layers continues to increase. Beyond a specific threshold, the benefits of adding more layers start to diminish due to the development of stress concentrations and interference between layers. As layers increase, the complexity of internal stress interactions grows, leading to stress localization in certain areas. This can reduce the panel's overall efficiency in distributing impact forces and may result in premature failure in localized regions. Additionally, the increased thickness from multiple layers can lead to a reduction in panel stiffness, making the structure more susceptible to buckling or delamination under impact, which compromises its energy dissipation capacity.

**Fig. 15:**
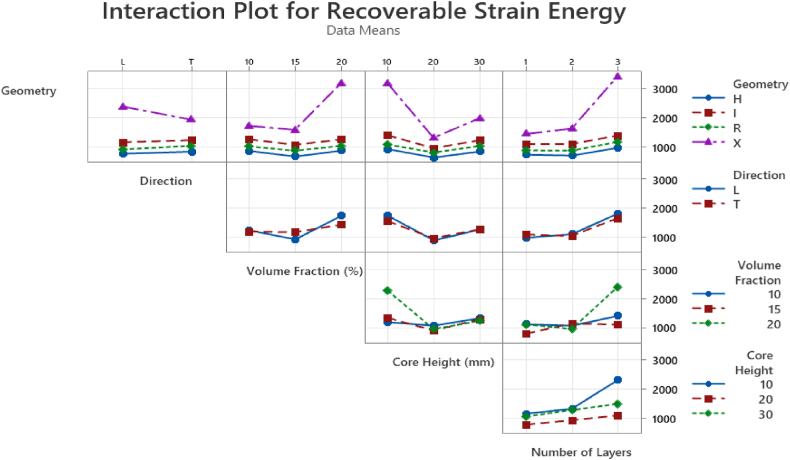
Interaction plot for recoverable strain energy.

The interaction plot in Fig. 15 shows the X-frame shows highest recoverable strain energy especially with the transverse orientation. As the volume fraction increases, the recoverable strain energy also increases regardless of core geometry or direction. The unit cell height effects the recoverable strain energy in different manners depending on the cell direction, however, the general trend suggests that at lower heights there is higher energy recovery.

Time perforation analysis plays an important role in understanding the effect of structural parameters on sandwich panel behaviour, particularly in terms of energy absorption and structural integrity. By examining the time, it takes for an impactor to perforate a sandwich panel, the panel's capacity to absorb and dissipate energy under impact can be assessed. Notably, a longer perforation time, given a fixed impact velocity, signifies enhanced energy absorption by the sandwich panel. In the analysis, von Mises stress contour plots were used to illustrate the time-dependent behaviour of the sandwich panels during impact.

**Fig. 16:**
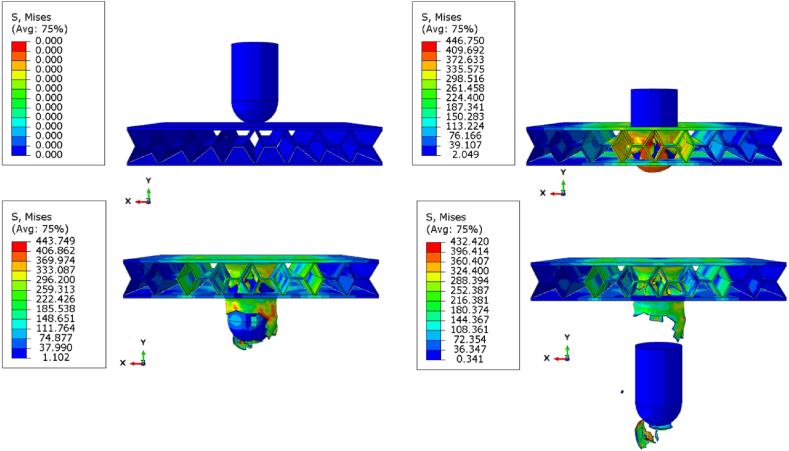
Contour plots for perforation of X-frame sandwich panels at (a) t = 0s; (b) t = 0.00625; (c) t = 0.0125; (d) t = 0.025.

Fig. 16 shows the von Mises stress distribution at various time intervals, providing insights on the stress evolution and structural response of the panel. The element registering the highest von Mises stress is observed at t = 0.00625s (Fig. 16b). The reported stresses exceed the yield stress of the constituent material, making them unsuitable for comparing peak stress differences. However, the trend in von Mises stress aligns with the expected softening due to local buckling as a consequence of the impact.

The reduction in the highest stress point can be attributed to complete perforation, where the impacted element is separated from the sandwich panel. Additionally, local buckling of cell walls can lead to redistribution of stress and a decrease in the overall stiffness of the structure, as damaged regions no longer contribute effectively to load bearing. This reduction in stiffness and the subsequent redistribution of stress results in a drop in the von Mises stress levels. Damage, particularly in the form of plastic deformation and fracturing, significantly reduces the elastic modulus of the material. As damaged areas no longer contribute to the panel's load bearing capacity, the stress carried by these regions decreases, which is reflected in the von Mises stress contours. Analysing these contour plots provides a deeper understanding of how different structural parameters influence the perforation process and the overall energy absorption capabilities of the sandwich panels. This understanding aligns with findings from similar studies in the literature [30] [31].

**Fig. 17:**
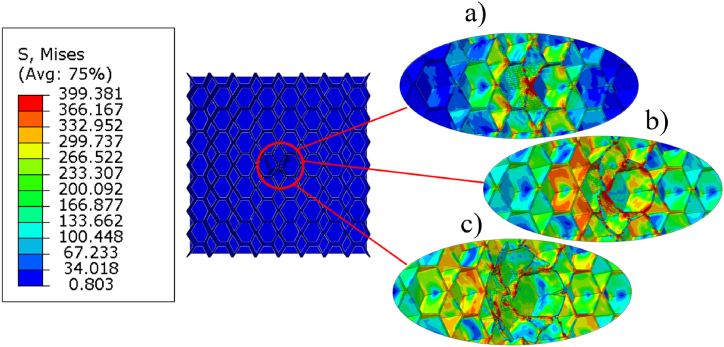
Stress contour plots illustrating top view on impact area of longitudinal unit cells (a) elastic deformation; (b) plastic buckling; (c) fracture of cell walls.

**Fig. 18:**
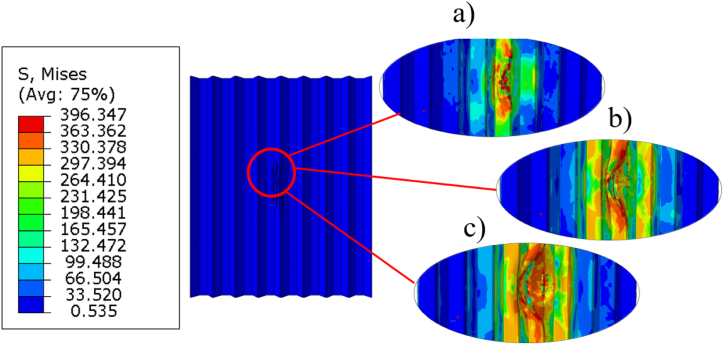
Stress contour plots illustrating top view on impact area of transverse unit cells (a) elastic deformation; (b) plastic buckling; (c) fracture of cell walls.

Fig. 17 presents stress contour plots, showing a top view of an X-frame core sandwich panel, with the top face sheet removed. Fig. 17a shows the initial impact at which the cells undergo elastic deformation without yielding since the impact force is still relatively low. As the impactor exerts greater force on the central 3x3 cells, buckling occurs, causing the middle unit cells to redistribute the stress to its neighbouring cells, resulting in plastic deformation (Fig. 17b). When the fracture stress is reached, stress distribution extends to the surrounding cells, indicating complete cell failure. The configuration of unit cells is longitudinal, impacting the cell walls around the void in the X-frame configuration, resulting in lower surface area for the impacted cell walls. In contrast, in the transverse direction (Fig. 18), higher surface area allows for more effective stress distribution to neighbouring cells. The recoverable strain energy of the higher surface area can be quite similar to that of the lower surface area (transverse vs. longitudinal). This can be attributed to the hollowness in the y-direction, which reduces recoverable strain energy. Consequently, the differences between longitudinal and transverse orientations become negligible when the volume fraction is equal in both directions, as these opposing effects tend to cancel each other out.

In the longitudinal orientation, the stress tends to be concentrated more heavily on the cell walls directly around the impact zone, as the smaller surface area makes it harder for the stress to distribute to neighbouring cells. This results in quicker onset of plastic deformation and potentially earlier cell failure due to localized stress concentration.

As for the transverse orientation, a higher surface area is available for distributing the impact forces. This leads to more effective stress redistribution, spreading the load more evenly across a larger number of neighbouring cells. As a result, the core is better able to absorb and distribute energy, which can delay the onset of plastic deformation and cell failure.

Additionally, the longitudinal orientation may exhibit quicker buckling and earlier plastic deformation of the impacted cells due to the smaller area for stress redistribution. Once buckling initiates, the force quickly transfers to neighbouring cells, but because of the limited number of cells involved, the structure may lose strength more rapidly. As for the transverse orientation, with a larger number of cells involved in the redistribution of stress, provides a delayed buckling response, improving the structure's ability to resist deformation longer and increasing the energy absorption capacity.

### 3.1 Regression analysis

Statistical analysis tools were employed to model the responses in terms of core topology, core volume fraction, core height, and number of layers on the extent of damage to the sandwich panel. Response surface methodology was used to construct a regression model that establishes a relationship between the response variables and the factors examined during simulation. For the model-fitting process, terms with high P-values in the analysis of variance were not considered, resulting in a model consisting of linear terms and their interactions. The resultant model demonstrates a reasonably accurate fit, with R-squared (R2) values of 78.02 %, and 66.12 % for the energy dissipated in damage and the total strain energy, respectively. Table 3, Table 4 present the regression equations (1), (2), (3) that elucidate how factors such as core height, volume fraction, number of layers, and core topology can influence damage on the sandwich panels. These regression equations were formulated in a complete linear format to express the response (R) as outlined in the equation below.

(2) $$R=a+b\left(Vf\right)+c\left(H\right)+d\left(N_{L}\right)+e\left(Vf^{2}\right)+f\left(H^{2}\right)+g\left(N_{L}^{2}\right)+h\left(Vf\right)\left(H\right)+i\left(Vf\right)\left(N_{L}\right)+j\left(H\right)\left(N_{L}\right)$$

where Vf, H, and N_L_ are the volume fraction (%), core height (mm), and number of core layers, respectively. The constant a and coefficients b, c, d, e, f, and g correspond to the linear and quadratic terms of these variables. Additionally, h, i. and j represent the interaction coefficients between volume fraction and core height, volume fraction and number of layers, and core height and number of layers, respectively. Table 3, Table 4, Table 5 provide the values for these terms for each response variable.

**Table 3:** Regression analysis equations: energy damage

Core Geometry	Direction	a	b	c	d
H-frame	Longitudinal	873	−31	−10	−484
I-frame	Longitudinal	1118	−32.7	−9.2	−451
Rhombus	Longitudinal	953	−29.5	−11.3	−479
X-frame	Longitudinal	1098	−26.9	−5.3	−449
H-frame	Transverse	657	−13.3	−9	−463
I-frame	Transverse	877	−15.1	−8.2	−430
Rhombus	Transverse	723	−11.8	−10.3	−459
X-frame	Transverse	880	−9.2	−4.2	−429

**Table 4:** Regression analysis equations: recoverable strain energy

Core Geometry	Direction	a	b	c	d
H-frame	Longitudinal	2284	−137	−61.6	214
I-frame	Longitudinal	2728	−139	−66.2	−631
Rhombus	Longitudinal	2329	−136	−60.4	−628
X-frame	Longitudinal	1107	8	−118.1	−657
H-frame	Transverse	2838	−163	−52.2	352
I-frame	Transverse	3283	−165	−56.8	−493
Rhombus	Transverse	2936	−162	−50.9	−490
X-frame	Transverse	1133	−18	−108.6	−519

**Table 5:** Optimization parameter

Response	Goal	Lower	Target	Weight	Importance
Energy Damage	Maximum	27.402	1099.7	1	1
Recoverable Strain Energy	Maximum	368.197	12619.2	1	1
Structure Mass	Minimum	–	0.1	1	1

The regression analysis equation for the energy dissipated by damage is:

(3) $$R=a+b\left(Vf\right)+c\left(H\right)+d\left(N_{L}\right)+0.251\left(Vf^{2}\right)+0.689\left(H^{2}\right)+92.4\;\left(N_{L}^{2}\right)+2.23\left(Vf\right)\left(H\right)+1.96\left(Vf\right)\left(N_{L}\right)+2\left(H\right)\left(N_{L}\right)$$

The regression analysis equation for the total recoverable strain energy is:

(4) $$R=a+b\left(Vf\right)+c\left(H\right)+d\left(N_{L}\right)+5.68\left(Vf^{2}\right)+4.47\left(H^{2}\right)+77\;\left(N_{L}^{2}\right)-6.01\left(Vf\right)\left(H\right)+50.5\left(Vf\right)\left(N_{L}\right)-18\left(H\right)\left(N_{L}\right)$$

Minitab was employed to determine the optimal design parameters that could simultaneously maximize energy damage and recoverable strain energy while minimizing the total mass of the structure. The Response Optimizer plot was utilized to achieve this goal. To identify the optimal mixture component that could cater to all objectives, energy damage, recoverable strain energy, and mass structure, specific goals for maximization or minimization were established using the available data, as outlined in Table 5.

**Fig. 19:**
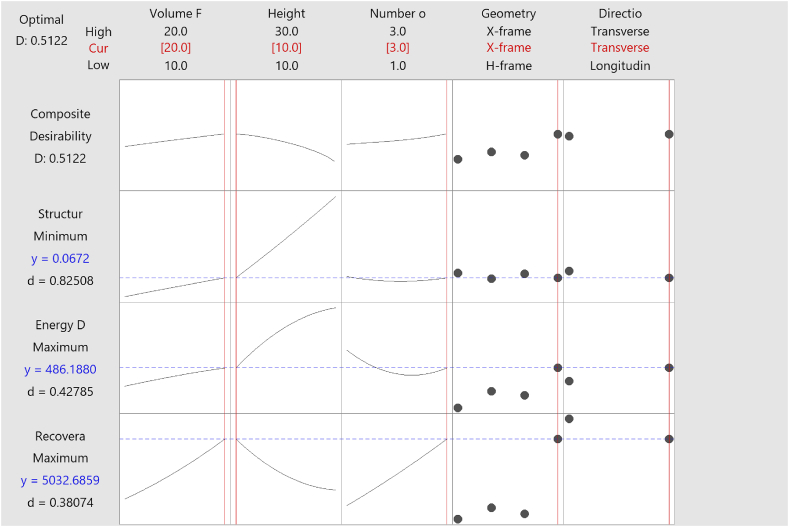
Response optimizer plot.

The optimal solution with the highest composite desirability (D = 0.5122) signifies the most favourable configuration for the specified optimization parameters. The response optimizer plot in Fig. 19 shows that the optimal configuration for maximizing energy damage and recoverable strain energy while minimizing the structure's mass is achieved with X-frame unit cells in the transverse direction, a core height of 10 mm is divided into 3 layers, and a unit cell volume fraction of 20 %. This configuration not only ensures high energy absorption and efficient stress distribution but also maintains a lightweight structure, making it suitable for applications requiring high impact resistance and structural efficiency.

The X-frame core demonstrated superior energy absorption capabilities in this study, particularly under low-velocity impact conditions, where it effectively distributed impact forces and minimized damage through its unique geometric configuration. While these results highlight the X-frame's excellent performance in low-velocity impact scenarios, it is important to consider whether this performance can be generalized to other structural applications.

The X-frame geometry is likely to offer significant benefits in applications where energy absorption is critical, such as in crashworthiness or blast protection, due to its efficient load distribution and ability to resist buckling. In these contexts, the X-frame's capacity to dissipate energy through plastic deformation could also be advantageous in high-velocity impacts or other dynamic loading situations.

For applications that involve static loads or different types of mechanical loading, such as compressive or tensile forces, the energy absorption advantage observed in low-velocity impacts may not directly translate to enhanced performance. In these cases, the X-frame core's structural efficiency and stiffness under static or quasi-static loading conditions would need to be evaluated separately. Moreover, the geometry of the X-frame may need to be optimized further for specific applications, as different loading scenarios could lead to different stress distributions and failure modes.

## 4 Conclusion

In this study, a comprehensive investigation into the intricate dynamics of sandwich panels subjected to low-velocity impact events was presented. The analysis encompassed both numerical simulations and experimental tests, aiming to focus on the role of various structural parameters in shaping the panels' behaviour and their energy absorption capabilities.

Using Abaqus/Explicit, detailed analyses were conducted to understand energy dissipation and recoverable strain energy. The energy dissipated in damage served as a measure of damage tolerance and impact resistance. The von Mises stress contour plots illustrated the time-dependent behaviour of the panels, highlighting stress evolution and structural responses. The total recoverable strain energy provides insights into how much energy is stored during deformation and indicates the panel's ability to endure and recover from impacts. Key findings include.

1 The X-frame core demonstrated the highest energy dissipation of 500 J, indicating superior load distribution and energy absorption capabilities. The transverse orientation further enhanced energy absorption (342 J) due to changes in impact area and inertia. Additionally, the X-frame core outperformed other geometries in recoverable strain energy, with a maximum of 2321 J. This high recoverable strain energy is attributed to the X-frame's higher bending stiffness and ductility, allowing it to endure higher strains before failure.
2 Increasing the volume fraction from 10 % to 20 % enhanced both energy dissipation and recoverable strain energy, showing a linear relationship with panel stiffness. However, higher volume fractions also introduced brittleness, indicating the need to balance volume fraction to optimize energy recovery.
3 Optimal energy absorption was achieved at a core height of 20 mm, beyond which further increases reduced stiffness and energy dissipation benefits. Lower unit-cell heights resulted in the highest recoverable strain energy, suggesting that shorter heights can lead to higher energy recovery. Conversely, increasing the number of core layers increased both energy dissipation and recoverable strain energy. However, the effects were non-linear due to the complexity of stress interactions within the layered structure, leading to stress concentrations and reduced efficiency.

Experimental tests on sandwich panels with different core shapes confirmed the numerical findings. Sandwich panels with X-frame cores demonstrated superior energy absorption, shown by the smallest impact area. Conversely, H-frame cores showed the largest damage area, indicating limited energy absorption capabilities. Statistical tools and regression models were employed to study the relationships between structural parameters and damage extent. The response surface methodology provided regression equations, revealing how factors such as core height, volume fraction, number of layers, and core topology influence damage. Minitab's response optimizer identified the optimal configuration—X-frame unit cells in the transverse direction, core height of 10 mm, divided into three layers, and a unit cell volume fraction of 20 %.

The X-frame configuration stands out as the most effective core geometry in this study, primarily due to its unique structural properties that enable superior energy absorption and efficient load distribution under low-velocity impact. The geometry of the X-frame features a network of shorter, angular cell walls arranged in a way that maximizes the load transfer capabilities from the point of impact to adjacent cells. This interconnected design allows the X-frame to handle stress more effectively compared to other geometries like the H-frame, I-frame, or Rhombus, which have less optimized stress distribution paths.

One of the key factors contributing to the X-frame's performance is its ability to prevent the rapid onset of plastic deformation. The configuration's unit cells provide a greater surface area for distributing the impact load, especially in the transverse direction, which means that the applied forces are spread over more cells, reducing localized stress concentrations that can lead to early cell failure. As a result, the X-frame can endure higher impact forces without suffering immediate damage, allowing for delayed plastic deformation and fracture of the cell walls. This is in stark contrast to other configurations, where stress tends to concentrate quickly in specific areas, leading to quicker buckling and perforation of the sandwich panel.

Furthermore, the X-frame demonstrates excellent energy absorption capacity. The force-displacement and energy dissipation results from the study show that the X-frame consistently dissipates more energy compared to the other geometries. The higher bending stiffness of the X-frame contributes to this, as it can bend and deform elastically for longer before reaching the failure point. This stiffness, coupled with a high recoverable strain energy, means that the X-frame can store and then release energy more effectively, enhancing the sandwich panel's overall impact resistance.

The insights from this study are significant for industries requiring lightweight, impact-resistant materials, such as aerospace and automotive sectors. The optimal design parameters ensure high energy absorption, efficient stress distribution, and lightweight structures, making these sandwich panels suitable for applications demanding superior impact resistance and structural efficiency.

## CRediT authorship contribution statement

**Assil Charkaoui:** Writing – original draft, Visualization, Methodology, Investigation, Formal analysis. **Noha M. Hassan:** Writing – review & editing, Supervision, Methodology, Investigation, Funding acquisition, Formal analysis, Data curation, Conceptualization. **Zied Bahroun:** Writing – review & editing, Supervision, Methodology, Investigation, Funding acquisition, Conceptualization.

## Declaration of competing interest

The authors declare that they have no known competing financial interests or personal relationships that could have appeared to influence the work reported in this paper.
